# AlgaePath: comprehensive analysis of metabolic pathways using transcript abundance data from next-generation sequencing in green algae

**DOI:** 10.1186/1471-2164-15-196

**Published:** 2014-03-14

**Authors:** Han-Qin Zheng, Yi-Fan Chiang-Hsieh, Chia-Hung Chien, Bo-Kai Justin Hsu, Tsung-Lin Liu, Ching-Nen Nathan Chen, Wen-Chi Chang

**Affiliations:** Institute of Tropical Plant Sciences, National Cheng Kung University, Tainan, 70101 Taiwan; Institute of Bioinformatics and Biosignal Transduction, National Cheng Kung University, Tainan, 70101 Taiwan; Institute of Marine Biology, National Sun Yat-sen University, Kaohsiung, 80424 Taiwan; Yourgene Bioscience Company Ltd, New Taipei City, 23863 Taiwan

**Keywords:** Algae, Pathway, Next-generation sequencing, Systems biology, Gene expression

## Abstract

**Background:**

Algae are important non-vascular plants that have many research applications, including high species diversity, biofuel sources, and adsorption of heavy metals and, following processing, are used as ingredients in health supplements. The increasing availability of next-generation sequencing (NGS) data for algae genomes and transcriptomes has made the development of an integrated resource for retrieving gene expression data and metabolic pathway essential for functional analysis and systems biology. In a currently available resource, gene expression profiles and biological pathways are displayed separately, making it impossible to easily search current databases to identify the cellular response mechanisms. Therefore, in this work the novel AlgaePath database was developed to retrieve transcript abundance profiles efficiently under various conditions in numerous metabolic pathways.

**Description:**

AlgaePath is a web-based database that integrates gene information, biological pathways, and NGS datasets for the green algae *Chlamydomonas reinhardtii* and *Neodesmus sp.* UTEX 2219–4. Users can search this database to identify transcript abundance profiles and pathway information using five query pages (Gene Search, Pathway Search, Differentially Expressed Genes (DEGs) Search, Gene Group Analysis, and Co-expression Analysis). The transcript abundance data of 45 and four samples from *C. reinhardtii* and *Neodesmus sp.* UTEX 2219–4, respectively, can be obtained directly on pathway maps. Genes that are differentially expressed between two conditions can be identified using Folds Search. The Gene Group Analysis page includes a pathway enrichment analysis, and can be used to easily compare the transcript abundance profiles of functionally related genes on a map. Finally, the Co-expression Analysis page can be used to search for co-expressed transcripts of a target gene. The results of the searches will provide a valuable reference for designing further experiments and for elucidating critical mechanisms from high-throughput data.

**Conclusions:**

AlgaePath is an effective interface that can be used to clarify the transcript response mechanisms in different metabolic pathways under various conditions. Importantly, AlgaePath can be mined to identify critical mechanisms based on high-throughput sequencing. To our knowledge, AlgaePath is the most comprehensive resource for integrating numerous databases and analysis tools in algae. The system can be accessed freely online at http://algaepath.itps.ncku.edu.tw.

**Electronic supplementary material:**

The online version of this article (doi:10.1186/1471-2164-15-196) contains supplementary material, which is available to authorized users.

## Background

The global energy crisis poses a major threat to human survival. Gradual depletion of non-renewable energy resources has increased energy costs and pollution. To remedy this situation, renewable energy sources such as biomass have been developed. Biomass is gathered from various plants (including crops or peas) and used to generate bio-alcohol or bio-fuel [1, 2]. However, using crops as a biomass source may crowd out food, especially in poor regions of the world [3]. Thus, an alternative species with less land requirement and high oil production needs to be identified. Algae are highly efficient in oil production, and are a promising biofuel source [4]. In addition to their possible use for biofuel, algae have many research applications [5]. For instance, because of its high species diversity, algae is an excellent model for evolution studies. The diversity of algae has been attributed not only to the evolution force, but also to horizontal gene transfer [6]. Allochthonous genes can increase species diversity and organism competition [6]. Further, because algae are capable of absorbing heavy metals, they can be used to remove pollution in waste water [7–11]. Currently, algae are the most important non-vascular model plants that are used for research.c Because of their diverse applications, numerous studies have investigated the functional genomics of algae. Therefore, with the increasing availability of algae genome and transcriptome data from next-generation sequencing, an integrated resource for the retrieval of gene expression data with metabolic pathways is essential for functional analysis in algae. Further, understanding cellular response mechanisms under different environments is a high priority in systems biology.

*Chlamydomonas reinhardtii* is a single-celled green algae, which is distributed worldwide in soil and water. *C. reinhardtii* is used mainly as a model organism when addressing fundamental issues such as photosynthesis, cellular movement, abiotic response mechanisms, and regulation of flagellar mechanism. This model organism is also an important model for non-vascular plant research and information on the genomics and transcript abundance of *C. reinhardtii* is available in public resources. *Neodesmus sp.* UTEX 2219–4 is a species of green microalgae, which was isolated from collection number UTEX 2219 from the University of Texas at Austin (UTEX) and identified subsequently as genus *Neodesmus*[12]*.* Wang *et al*. [12] indicated that oil bodies were accumulated significantly under nitrogen starvation and osmotic stress in *Neodesmus sp.* UTEX 2219–4. Therefore, this strain was identified as having a high potential for biofuel applications. This study analyzed the transcriptome deep sequencing data that was obtained from four samples under various conditions.

Increasing amounts of next generation sequencing (NGS) data have become available in recent years and algal biochemistry and biology have attracted growing interest for their potential in developing renewable biofuel. In addition, RNA-seq technology has been used widely to identify algal cellular physiology and metabolism under various types of abiotic stress and/or nutrient deficiency [13–18]. For example, a previous study investigated cellular response mechanisms by characterizing the *C. reinhardtii* transcriptome under nutrient-replete and sulfur-depleted conditions [16]. Based on Roche 454 and Illumina sequencing, Miller *et al.*[17] investigated diversion of metabolism and transcript abundance during nitrogen repletion and deprivation in *C. reinhardtii*. Consequently, the increasing use of NGS has made the integration of high-throughput data from different experiments a priority concern. Genevestigator [19] is a high performance platform that integrates many public microarray experiments and visualizes gene expression data in different biological contexts. However, although nine important higher plants have been collected in the Genevestigator database, none of them are non-vascular plants. The Bio-Analytic Resource for Plant Biology (BAR) [20] also integrates numerous microarray data and provides expression profile similarity rankings of homologous genes in plant species; however, BAR cannot access algae-related information. BioCyc [21] is a collection of databases that provides the metabolic pathways of sequenced organisms including *C. reinhardtii*, but no gene expression data have been integrated into the collection. To our knowledge, the Algal Functional Annotation Tool (AFAT) is the first database in which algae gene expression data and metabolic pathways have been collected [22]. AFAT provides an integrated data-mining environment for algal genomics by integrating multiple annotation databases into a centralized system. Unfortunately, the metabolic pathways and gene expression profiles are displayed in separate windows in AFAT, making it difficult for users to understand the variations in cellular responses under different conditions. Additionally, the complex IDs and datasets in AFAT can often confuse users who want to search for interesting genes and pathways. For gene expression analysis, the AFAT website provides only an expression similarity search and the result page outputs only a Gene ID. As a result, it is relatively difficult to compare the variations of gene expression under different conditions using AFAT tools. Surprisingly, there are currently no other databases or tools that can be used to combine gene expression data with metabolic pathways in algae for systems biology, thereby necessitating the development of additional databases and related tools.

In this work, the novel AlgaePath database, which allows the efficient retrieval of cellular response data from different conditions, was developed. AlgaePath integrates various high-throughput datasets of *C. reinhardtii* and the stress-induced oil accumulation dataset of *Neodesmus sp*. UTEX 2219–4. AlgaePath can be used to identify transcript abundance profiles and to compare variations among different conditions in a pathway map through five query functions (Gene Search, Pathway Search, Differentially Expressed Genes (DEGs) Search, Gene Group Analysis, and Co-expression Analysis). The novel database can be accessed freely online at http://AlgaePath.itps.ncku.edu.tw.

## Construction and content

**Figure 1:**
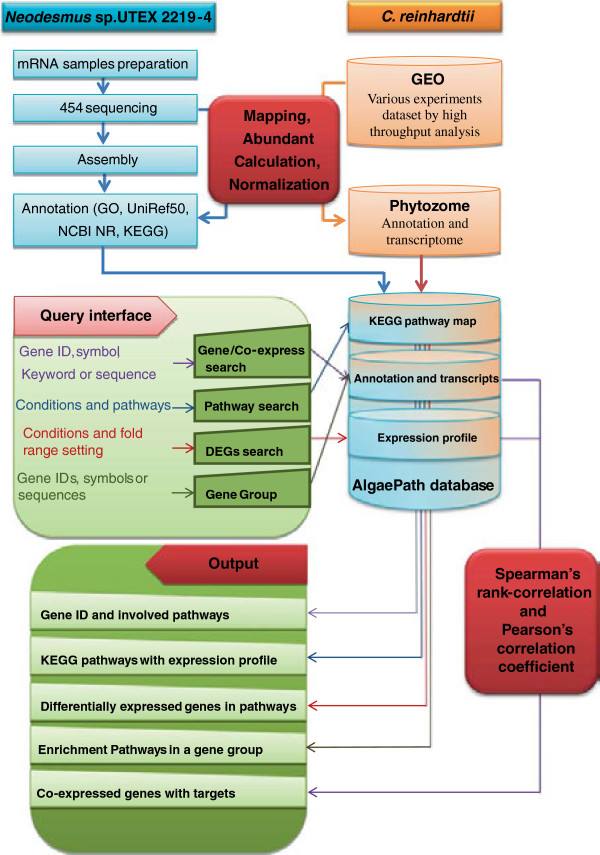
**The flowchart of AlgaePath.**

AlgaePath is a web-based database, which includes a high-throughput experimental dataset and information for *C. reinhardtii* and *Neodesmus sp.* UTEX 2219–4. The flowchart of AlgaePath is displayed in Figure 1. The system runs on an Apache web server on a Linux operating system. All the data are stored in a MySQL database and displayed in web pages, which are written in the PHP, Perl, and HTML programming languages. Details of the AlgaePath data and structure are described below.

### Transcriptome data of *Neodesmus sp.*UTEX 2219–4

The *Neodesmus sp*. UTEX 2219–4 transcriptome was sequenced on a Roche 454 system. The transcriptome data were obtained under four conditions (normal, nitrogen starvation, sorbitol stress, and salt stress) and have been integrated into the AlgaePath database. The sequence data processing and analysis have not been published (unpublished data in Dr. Ching-Nen Nathan Chen’s lab). The assembled sequences were annotated based on their similarity to sequences in the NCBI non-redundant (nr) database and the UniProt UniRef50 protein database. Currently, there are 20,698 *Neodesmus sp.* UTEX 2219–4 transcripts (including isotigs, contigs, and singletons) in the AlgaePath database.

### Transcriptome data of *C. reinhardtii*

The *C. reinhardtii* reference sequences and their annotation were downloaded from Phytozome (http://www.phytozome.net/, v5.3.1 of *Chlamydomonas* annotations) [23]. A total of 19,529 transcripts with annotations from Pfam (10,122) [24], PANTHER (8,905) [25], NCBI eukaryotic orthologous groups (KOG) (5,623) [26], KEGG (2,724 KEGG ortholog), and *Arabidopsis* homologous genes (9,106) were obtained. The RNA-seq transcriptome data were accessed from the Gene Expression Omnibus (GEO). In this study, the GSE17970, GSE24367, GSE25622, GSE33927, GSE33548, GSE34826, and GSE35305 datasets were used [13–18]. The conditions used for RNA-seq transcripts are sulfur depletion, nitrogen deprivation, mineral nutrient treatment, various concentration of carbon dioxide (CO_2_), oxidative stress, and Fe deprivation, including 45 samples. Adaptors were trimmed from all the sequences in the different samples and then the reads were mapped back to the reference sequences. The Bowtie2 [27] and BLAT [28] programs were used with their default parameters to map the Illumina sequence reads (Solexa) and the 454 pyro-sequence reads separately to the reference sequences. Finally, the expression level of each of the transcripts was calculated using reads per kilobase per million reads (RPKM).

### Normalization of expression data of each transcripts and identification differentially expressed genes

Because different transcriptome datasets were used, Deseq R package was applied to normalize various algae raw data and identify differentially expressed genes (DEGs) depending on negative binomial distribution. DEGs based on 17 comparison groups (see Additional file 1: Table S1) were pre-run and saved in AlgaePath database. However, there is the other option in AlgaePath web interface. Various samples could be selected to compare gene expression level, and identify DEGs based on expression fold change. Furthermore, Giorgi et al. indicated that Variance-Stabilizing Transformated (VST) RNA-seq data brings RNA-seq samples hierarchically closer to microarrays than RPKM normalization or raw counts when reconstructing co-expression networks [4]. Therefore, Variance-Stabilizing Transformation (VST) method was used to transform DEseq pre-normalized data. The VST was also performed by Deseq R package [29]. The VST normalized data were than used to analyze co-expression genes.

### Analysis of co-expressed genes

For the co-expression analysis, all the samples (excluding mutant samples) were divided into six categories (all conditions (GSE17970, GSE24367, GSE25622, GSE33927, GSE33548, GSE34826, and GSE35305), nitrogen deprivation (GSE24367), mineral nutrient treatment (GSE17970, GSE25622), carbon dioxide treatment (GSE33927), oxidative stress (GSE34826), and iron deprivation (GSE35305)). Based on Pearson’s correlation coefficient (PCC), the similarity of expression patterns across various samples in a category was measured, which represents the co-expressed level between a pair of genes. The co-expressed genes were then calculated, depending on the different categories. Next, the gene-pairs with PCC values (r) between -0.5 and 0.5 were removed from the co-expression data. Finally, the 100 best positive and 100 best negative correlations of a transcript were stored in the AlgaePath database. The PCC values were calculated as:


*and**denote the average expression values of X and Y genes under all conditions in each category, respectively n represents the total number of samples in each category.*

Additionally, The Spearman’s rank-correlation is less sensitive than the Pearson correlation to strong outliers. AlgaePath also provide the co-expression results using Spearman’s correlation. Users can identify co-expression genes based on different statistics methods.

Pathways identification of each transcript and gene group analysis.

KEGG genes and KEGG orthology (KO) were downloaded from the KEGG database (Release 2013) [30]. The KO IDs were mapped to transcripts annotated in AlgaePath. Related pathways of each transcript were then identified using the KEGG pathway reconstructed tool. For gene group analysis, pathway enrichment in a group of gene sets was analyzed using the hypergeometric distribution method [31]. The number of transcripts involved in each pathway was calculated as the abundance pathways in a group of gene sets. Pathway enrichment was then verified based on the p-value of each pathway following the hypergeometric distribution method as follows:


where, *i* denotes the number of transcripts in the gene group involved in the *j* pathway; *n* is the total number of transcripts in the gene group annotated in KEGG; *M* is the number of all *C. reinhardtii* transcripts involved in the *j* pathway; and *N* is the total number of all *Ch. reinhardtii* transcripts annotated in KEGG.

## Utility and discussion

### Web interface of AlgaePath

**Figure 2:**
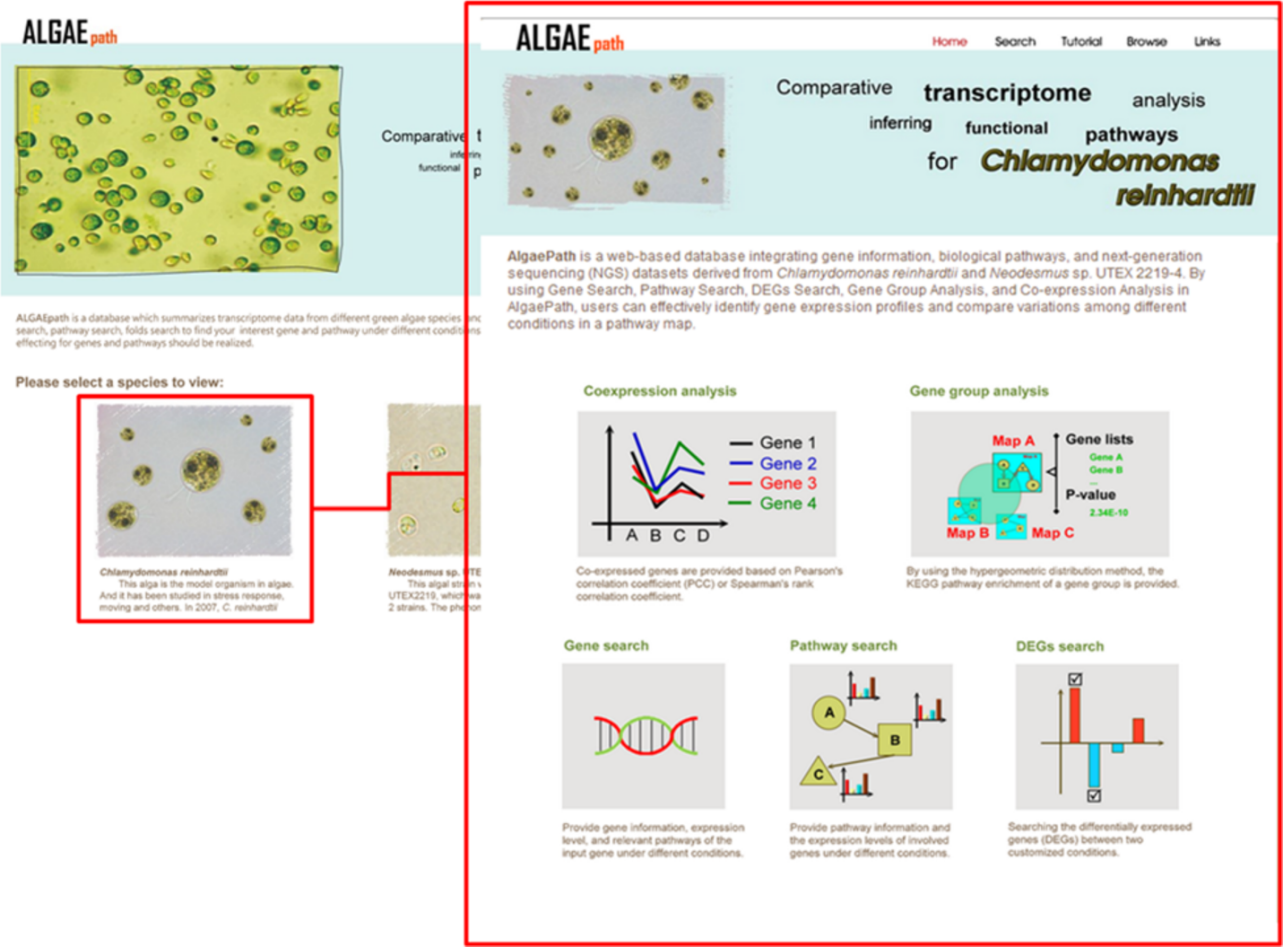
**The web interface of AlgaePath.**

**Figure 3:**
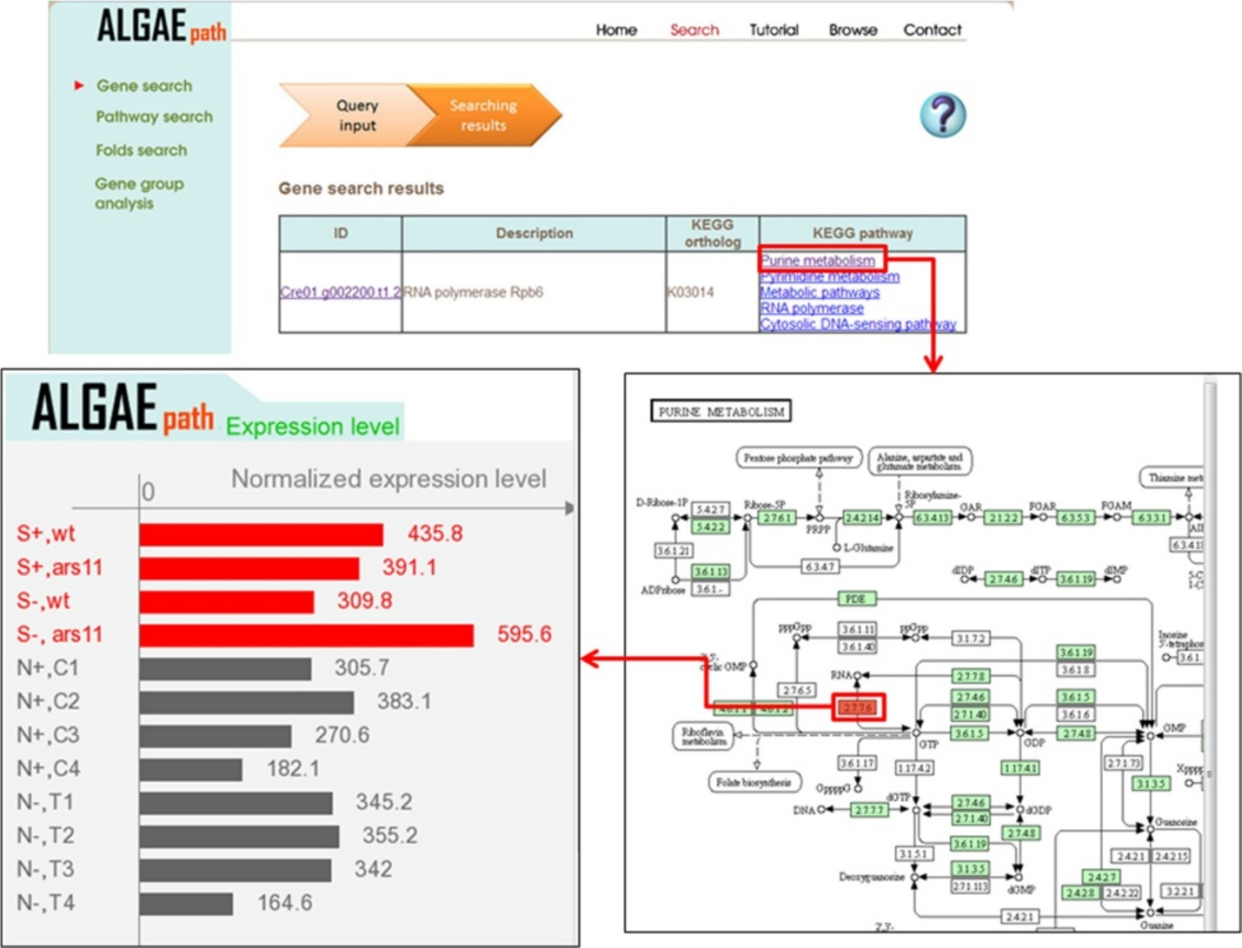
**The output result of “Gene Search” in AlgaePath.** The input gene is marked in red background, and the gene expression levels under different conditions are displayed in a popup window.

**Figure 4:**
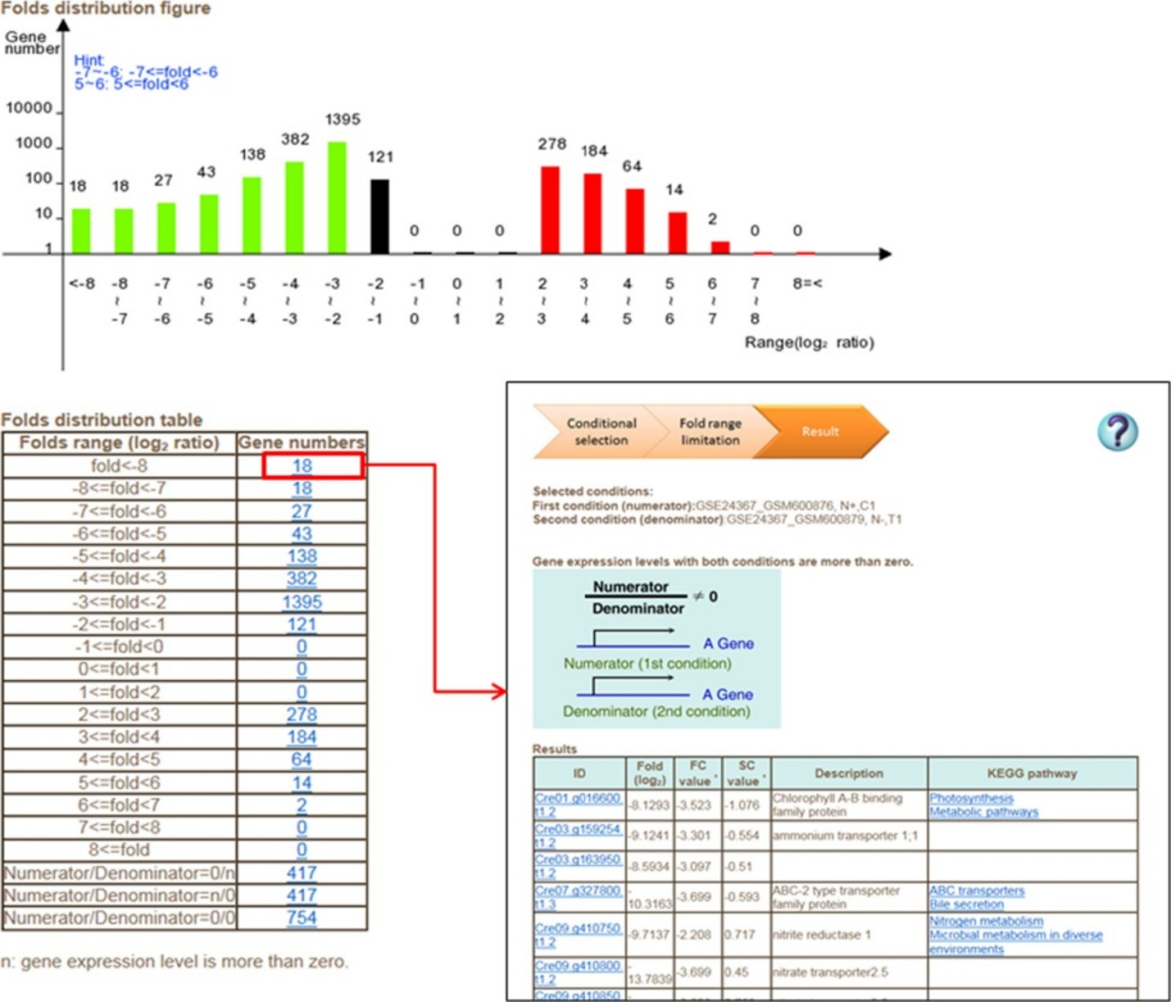
**The output results of “Differentially Expressed Genes (DEGs) Search” in AlgaePath.** The statistics numbers of genes in particular folds ranges are listed in a table. The further information about those genes could be retrieved by clicking the gene numbers on the web page.

**Figure 5:**
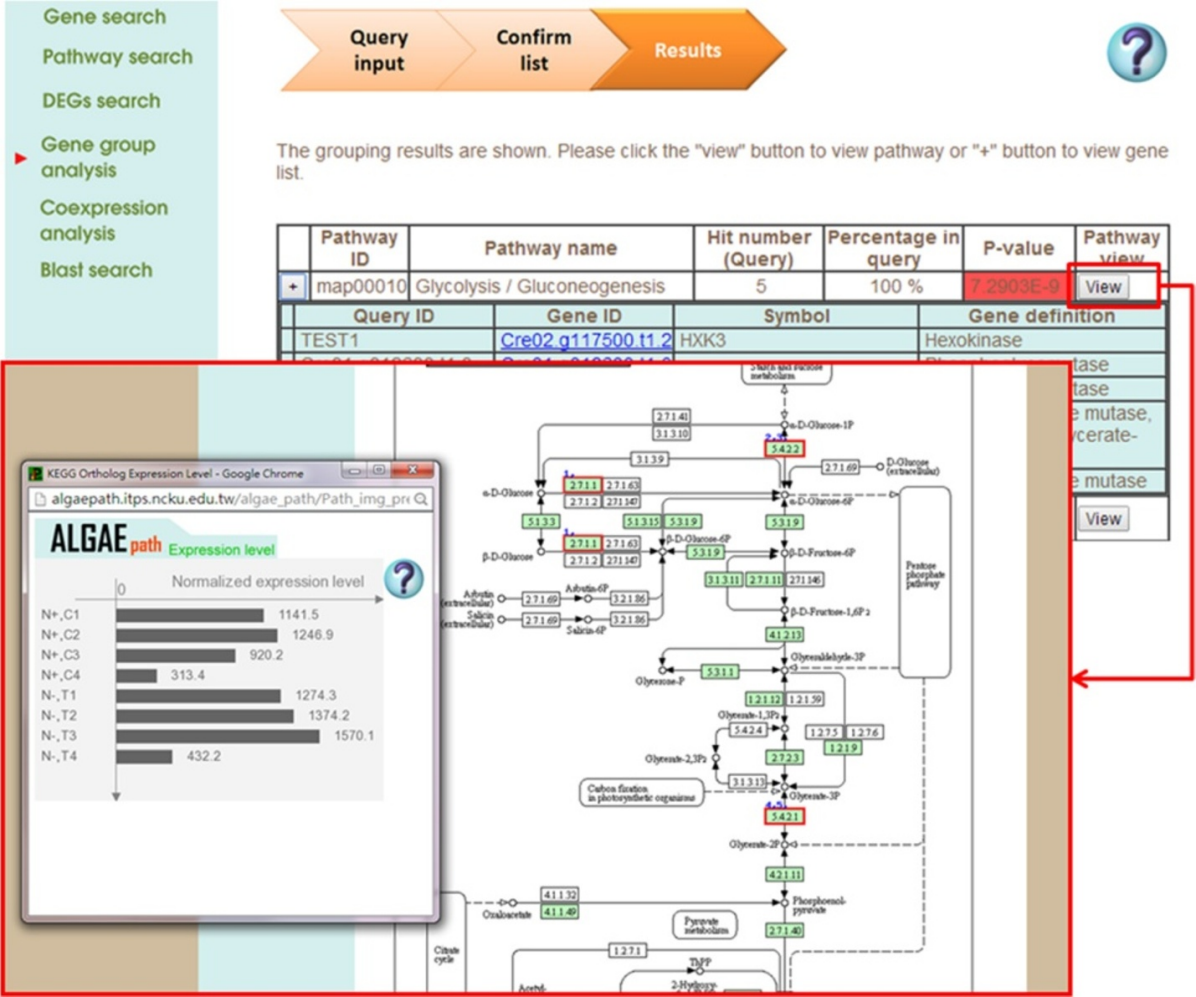
**The output results of “Gene Group Analysis” in AlgaePath.** The pathways related to a group of genes are listed in a table with hit percentage and p-value. After clicking pathway viewer, query genes will be marked in red rectangle in a pathway map. The gene expression profile of each gene will be displayed in a pop-up window.

**Figure 6:**
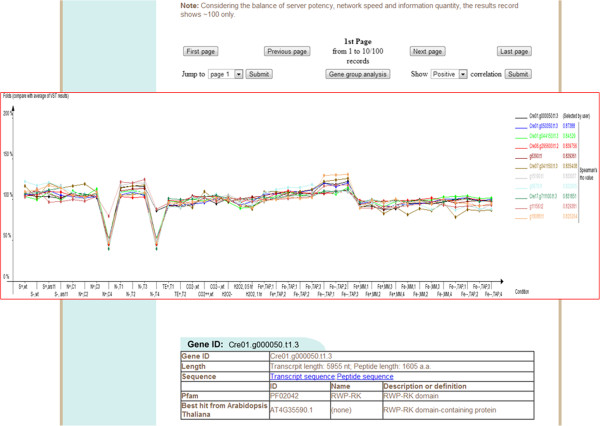
**The output results of “Co-expression Analysis” in AlgaePath.** 10 correlation genes of the query gene will be displayed in one output page. Totally, 100 positive and negative correlation genes could be accessed, respectively.

Two species (*C. reinhardtii* and *Neodesmus sp*. UTEX 2219–4) are displayed on the AlgaePath home page (Figure 2). By clicking on either of the species (for example *C. reinhardtii*), five transcriptome analysis functions are displayed; namely, Gene Search, Pathway Search, Differentially Expressed Genes (DEGs) Search, Gene Group Analysis, and Co-expression Analysis. Tutorial and Browse options are displayed at the top of the home page for each species. On the Gene Search page, a gene symbol, keyword, gene ID from various databases (Pfam, PATHER, KEGG K ENTRY, and KOG), DNA sequence, or protein sequence can be input as a query. Gene Search uses the query to retrieve the gene expression profiles in a specific pathway under different conditions (Figure 3). The Pathway Search page allows users to first select the stress conditions of interest. This opens the pathway browser in which either one of the listed pathways can be selected or a pathway search can be performed using a keyword. The algae genes (marked in green) involved in the pathway are then displayed in a pathway figure. Clicking on an identified gene opens a link to the KEGG ortholog information for that gene. Expression level profiles under various conditions and information about the gene are also displayed. The DEGs Search page has two options: “Analyzing by Deseq R package” and “Fold change search”. DEGs from 17 comparison groups can be identified by using “Analyzing by Deseq R package”, respectively. “Fold change search” can be used to compare differentially expressed genes between two conditions that can be selected by the user. The fold range can be chosen and/or keywords can be used to limit the number of results. The search results pages are similar either use “Analyzing by Deseq R package” or “Fold change search”. The numbers of genes within the chosen fold range or p-value are displayed both as a graph and in a table. Detailed information about the genes and pathways with a particular fold change can be accessed by clicking on the gene numbers in the table (Figure 4). The Gene Group Analysis page provides a tool for users to perform pathway enrichment analysis for a group of input genes. Pathways related to the user-selected group of genes are listed in a table which also displays a hit percentage and p-value (Figure 5). The pathway view button can be used to display the transcript abundance levels of these genes under numerous conditions. The Co-expression Analysis page can be used to retrieve gene information and expression profiles of genes that have similar expression profiles (based on PCC or Spearman’s correlation) to a user-input gene. First, the condition of interest and the gene of interest are selected. Then co-expressed genes under a particular experiment are identified and top ten correlated genes for each query gene are displayed. The functional related genes with similar expression profiles across different experimental conditions can be accessed directly (Figure 6). Up to 100 positive and negative correlations can be accessed from different pages. Co-expression analysis can also be accessed from the Gene Search page.

### Comparative analysis of gene expression profiles under various conditions

Comparative analysis of gene expression under various conditions can help biologists identify critical genes involved in changes of interest. For example, the feasibility of using algae in biofuel production has been studied [4] and scientists have attempted to overexpress acetyl-CoA carboxylase (a substrate for fatty acid production) in microalgae to improve oil production [29]. Unfortunately, until now, the performance of algae in oil production has been insignificant [32]. Rismani-Yazdi *et al.*[33] recently identified some important metabolic pathways related to microalgae-based biofuel feedstock by comparing transcriptome sequencing data under four different conditions. Numerous NGS transcriptome datasets are currently available in public resources and they need to be integrated so that important checkpoints can be identified in biological mechanisms. Importantly, in the AlgaePath database, users can easily investigate variations in transcript abundance under various conditions in a specific pathway based on user selection. All output result pages are displayed in a figure and in a downloadable table, as shown in Figure 3. The user-friendly interface can help users determine efficiently whether their genes of interest undergo significant changes in expression under different conditions. In this sense, AlgaePath is similar to the other resources that have been developed for higher plants (e.g. Genevestigator and the BAR database). Importantly, the biological pathways in AlgaePath are thoroughly combined with the transcript abundance data. Many important phenotypic changes that cause serious biological reactions are affected by more than one gene; therefore, researchers need to determine which genes have expression patterns that are similar to their target gene. Such information can help biologists clarify the mechanisms that they are interested in. Because co-expression analysis is very important in systems biology research, the ability to study the co-expression of genes in the six categories available in AlgaePath will be highly effective. The six categories were designed to provide users with a variety of options for data mining because many genes are co-expressed only under specific conditions. The AlgaePath system will provide users a multifunctional analysis platform for algae-related research.

### Case study: identification of critical genes during nitrogen starvation and sulfur depletion

**Figure 7:**
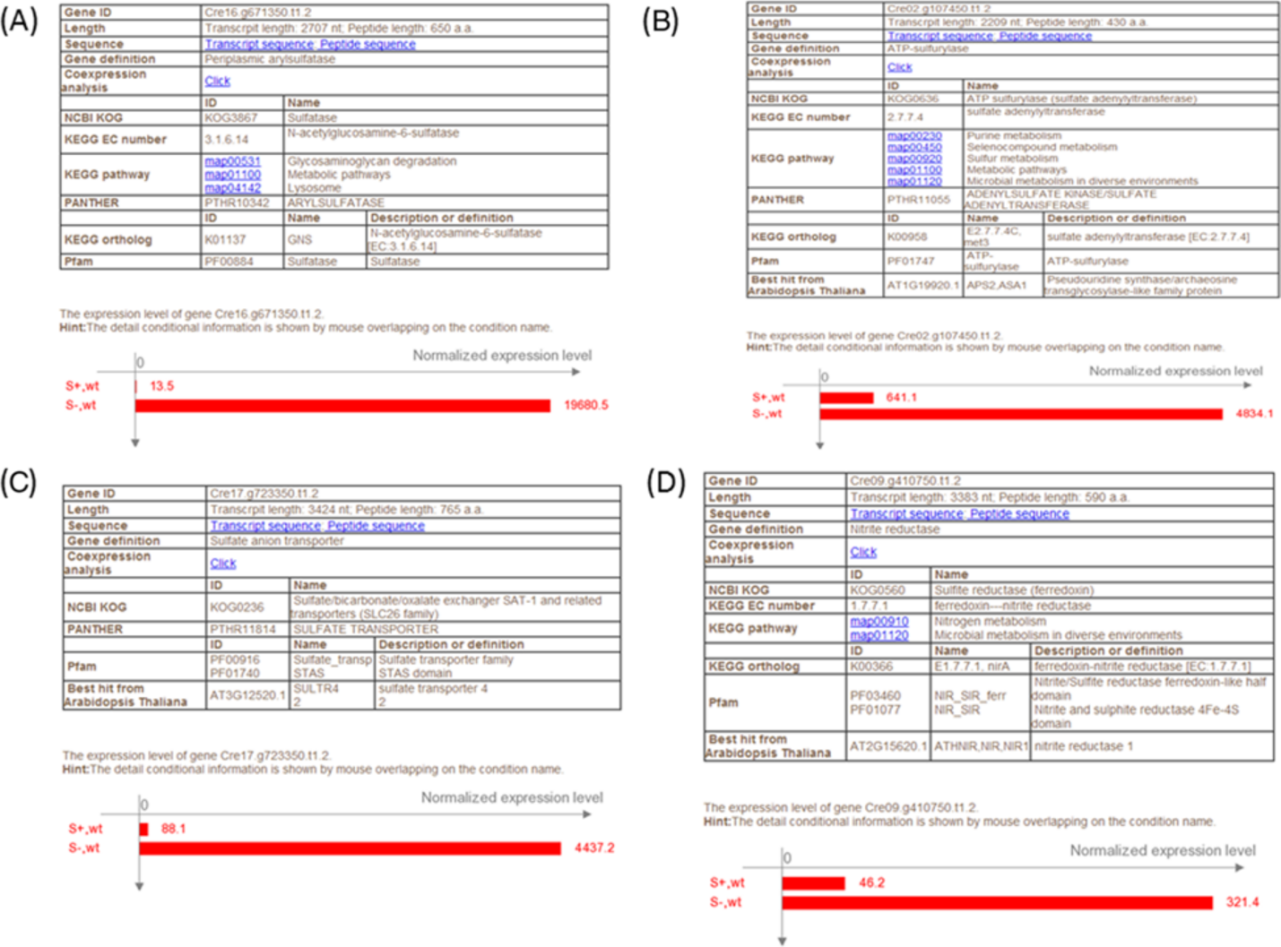
**A case study result: the gene expression levels changed during sulfur starvation. (A)** Arylsulfatase (Cre16.g671350.t1.2), **(B)** ATP-sulfurylase (Cre02.g107450.t1.2), **(C)** Sulfate transporter (Cre17.g723350.t1.2), and **(D)** Sulfite reductase (Cre09.g410750.t1.2).

The accumulation of oil bodies in *C. reinhardtii* under nitrogen starvation conditions was reported previously [34]. Several up-regulated and down-regulated genes in lipid biosynthesis pathway under nitrogen starvation conditions were identified using the AlgaePath database [17]. For instance, the diacylglycerol O-acyltransferase (DGAT, Cre12.g557750.t1.3), fatA acyl-ACP thioesterase (Cre06.g256750.t1.2), and plant stearoyl-acyl-carrier-protein desaturase family protein (Cre17.g701700.t1.2 and g17011.t1) genes were found to be significantly up-regulated under nitrogen starvation using the DEGs Search tool in AlgaePath. In addition, the variation of the expression profiles of genes involved in the fatty acid biosynthesis pathway were identified easily using Pathway Search. Sulfur is essential in the synthesis of proteins, lipids, and various metabolites. Because most organisms, including *C. reinhardtii*, have limited sulfur storage abilities, the continuous uptake of sulfur from the surrounding environment is critical to their survival. Based on microarray experiments, Zhang *et al.*[35] identified the responses of numerous genes to sulfur depletion. The identified genes were involved in sulfur metabolism, photosynthesis, carbon metabolism, respiration alternative electron transfer pathways/ATPase/transporters, oxidative stress, chaperones, proteolysis, signal transduction, and transcription, and, in particular, several genes related to sulfur metabolism or biosynthesis were significantly induced during sulfur starvation (e.g., arylsulfatase (ARS), ATP-sulfurylase, sulfate transporter, and sulfite reductase) [35]. These genes were validated using the AlgaePath system because the output results demonstrated that the transcript abundance levels increased obviously under sulfur depletion. Based on the AlgaePath data, the folds change between the sulfur starvation treatment and control samples, ARS (Cre16.g671350.t1.2), ATP-sulfurylase (Cre02.g107450.t1.2), sulfate transporter (Cre17.g723350.t1.2), and sulfite reductase (Cre09.g410750.t1.2) genes was found to be up-regulated by more than 2 folds (P-value < 0.01) (Figure 7). These results suggest that AlgaePath can accurately represent phenomena reported in previous studies and can be used to identify the responses of some important genes to environmental stresses.

### Evaluation of AlgaePath and future perspectives

The Algal Functional Annotation Tool (AFAT) integrates many algae databases for use in algae research [22]. AFAT has large-scale analysis functions, including pathway enrichment analysis and differential expression analysis; however, despite its capability for processing high-throughput or microarray results, AFAT has a number of limitations. For example, most of the query tools in AFAT require specific gene IDs from specific data sets (Augustus u10.2, Augustus v5.0, JGI v3.0, JGI v4.0) and no gene symbols or sequences are allowed as input. Further, transcript abundance levels and pathways in AFAT are presented on separate result pages, which makes it difficult for users to evaluate expression patterns under various conditions in a pathway. Therefore, in this work a user-friendly, web-based database for algae was developed. The results of a comparison between the AFAT and AlgaePath characteristics are shown in Table 1. A number of different query types are available in AlgaePath, and the transcript abundance data under different conditions are displayed simultaneously in a pathway map. Broadly, AlgaePath can be used to retrieve a cellular response in a pathway under a specific condition. Identifying differentially expressed genes between various samples is crucial to elucidating biological response mechanisms under a particular condition. Notably, the AlgaePath system helps users find critical genes related to cellular responses relatively easily. The ability to search for similar expression profiles is usually necessary to investigate the function of a novel gene. Both AFAT and AlgaePath can be used to identify co-expressed genes; however, in AFAT, determining gene functions based only on a gene ID can be rather challenging. To retrieve gene functions in AFAT, users have to access JGI (http://genome.jgi-psf.org/) online by clicking each ID number. On the other hand, in AlgaePath it is relatively easy to study genes that are positively and negatively correlated with the query gene, and all gene lists and information are downloadable from the web site.

**Table 1:** **The comparison between algal functional annotation tool (AFAT) [** [22] **] and AlgaePath**

Content	AFAT	AlgaePath
Species	*Chlamydomonas reinhardtii*	*Chlamydomonas reinhardtii*
	*Chlorella* NC64A	*Neodesmus sp.* UTEX 2219-4
Search interface	Identifiers ID and keyword	Gene symbol, Gene ID from various database, keyword, DNA/protein sequences
Pathways information of each transcript	Yes, only pathway map (out link to KEGG)	Yes, combine pathway map with transcript abundance profiles under various conditions
Gene group analysis (Pathway enrichment)	Yes, only mark genes in a pathway map (out link to KEGG)	Yes, not only mark genes in a pathway map but with transcript abundance profiles under various conditions
Differentially expression genes	No	Yes, easily identify differentially expressed genes between two samples
Expression similar search	Yes, only provide identifier ID in the expression map	Yes, provide detail information of co-expression genes
Pathway map with gene expression profiles	No	Yes

A major advantage of AlgaePath over AFAT lies in its ability to integrate transcript abundance and pathway maps; however, although the genome sequence of *C. reinhardtii* was completed in 2007, the functions of many of the genes are still unknown [36]. In addition, some of the algal gene information in AlgaePath is limited; therefore, as more high-throughput experimental datasets become available it will be essential to continuously update the high-throughput data in AlgaePath. Although single datasets can be used to find interesting phenomena and to solve some related problems, the integration of many datasets can help increase the ability of researchers to identity variations in cellular responses under various conditions. Therefore, an improved normalization method needs to be developed to normalize many datasets from various platforms, including microarray, and Roche 454 or Illumina.

## Conclusions

The emergence of whole transcriptome and genome research has made an integrative database for displaying cellular response essential. Algae are important non-model plants that have many research applications, including higher species diversity, sources of biofuel, adsorption of heavy metals and, following processing, health supplements. AlgaePath is a web-based database that comprehensively integrates *C. reinhardtii* and *Neodesmus sp.* UTEX 2219–4 gene information, biological pathways, and transcript abundance profiles from various databases. AlgaePath provides an effective interface for users interested in obtaining further insights into the transcript response mechanisms in different metabolic pathways under various conditions. Moreover, the Gene Group Analysis and Co-expression Analysis tools in AlgaePath can be used to detect similar expression transcripts of a target gene and enrichment pathways (functions) in a gene group. The results obtained using AlgaePath will provide a valuable reference for future efforts to elucidate critical mechanisms by mining high-throughput data. Importantly, the AlgaePath database is a significant contribution to algae research.

## Availability and requirements

The AlgaePath database is publicly available at http://AlgaePath.itps.ncku.edu.tw.

## Electronic supplementary material

Additional file 1: Table S1: The comparison groups for differentially expressed genes (DEGs) identification in AlgaePath. (DOCX 17 KB)
