# Supplementary material for: AlgaePath: comprehensive analysis of metabolic pathways using transcript abundance data from next-generation sequencing in green algae
Source: BMC Genomics. 2014 Mar 14;15(1):196. doi: 10.1186/1471-2164-15-196 (PMC4028061; doi:10.1186/1471-2164-15-196)
Supplement: Supplementary file 1 — Additional file 1: Table S1: The comparison groups for differentially expressed genes (DEGs) identification in AlgaePath. (DOCX 17 KB) [file 12864_2013_7034_MOESM1_ESM.docx]

Table S1. The comparison groups for differentially expressed genes (DEGs) identification in AlgaePath

| groups for comparison |
| --- |
| **Sulfur deplete**   1. wild type vs. SNRK2.1 mutant (control) 2. wild type vs. SNRK2.1 mutant (treatment) 3. wild type (control) vs. wild type (treatment) 4. SNRK2.1 mutant (control) vs. SNRK2.1 mutant (treatment) |
| **Nitrogen deprivation**   1. control vs. treatment |
| **Revised trace element recipe**   1. control vs. treatment |
| **CO_2_ concentration**   1. wild type vs.cia5 mutant (very low co_2_ concentration) 2. wild type vs.cia5 mutant (low co_2_ concentration) 3. wild type vs.cia5 mutant (high co_2_ concentration) 4. wild type (very low CO_2_ concentration) vs. Wild type (high CO_2_ concentration) 5. cia5 mutant (very low CO_2_ concentration) vs. cia5 mutant (high CO_2_ concentration) |
| **Oxidative stress**   1. wild type vs. mutant (Singlet oxygen-mediated stress) 2. control vs. treatment (Hydrogen peroxide stress) |
| **Fe deprivation (TAP media)**   1. control vs. treatment (Fe repletion vs. Fe deficient) 2. control vs. treatment (Fe repletion vs. Fe limitation) |
| **Fe deprivation (Minimal media)**   1. control vs. treatment (Fe repletion vs. Fe deficient) 2. control vs. treatment (Fe repletion vs. Fe limitation) |
